# Evaluating the Long-Term Impact of Cytoreductive Surgery for Gastric Cancer with Peritoneal Metastasis: Are We on the Right Path?

**DOI:** 10.3390/jpm15070300

**Published:** 2025-07-10

**Authors:** Cecilia Orsini, Matteo Aulicino, Giorgio D’Annibale, Marianna Cantelmo, Sara Totaro Aprile, Paolo Catania, Lorenzo Barberis, Federica Ferracci, Miriam Attalla El Halabieh, Carlo Abatini, Claudio Lodoli, Andrea Di Giorgio, Antonia Strippoli, Fabio Pacelli, Francesco Santullo

**Affiliations:** 1 Department of Surgical Sciences, Catholic University of the Sacred Heart, 00168 Rome, Italysara.totaroaprile01@icatt.it (S.T.A.); paolo.catania01@icatt.it (P.C.); federica.ferracci@guest.policlinicogemelli.it (F.F.); fabio.pacelli@policlinicogemelli.it (F.P.); 2 Surgical Unit of Peritoneum and Retroperitoneum, Fondazione Policlinico Universitario A. Gemelli IRCCS, Largo Agostino Gemelli 8, 00168 Rome, Italyfrancesco.santullo@policlinicogemelli.it (F.S.); 3 Comprehensive Cancer Center, UOC Medical Oncology, Fondazione Policlinico Universitario Agostino Gemelli IRCCS, 00168 Rome, Italy

**Keywords:** HIPEC, cytoreductive surgery, gastric cancer, peritoneal carcinomatosis, oncological outcomes

## Abstract

**Background:** Peritoneal metastases from gastric cancer (GCPM) represent a significant clinical challenge in terms of therapeutic options and prognosis. Cytoreductive surgery (CRS) combined with hyperthermic intraperitoneal chemotherapy (HIPEC) has demonstrated promising survival benefits within a multimodal approach, particularly in carefully selected patients. **Methods:** This retrospective single-center study evaluated outcomes in patients with synchronous GCPM treated with CRS + HIPEC following neoadjuvant chemotherapy. The primary endpoints included overall survival (OS), disease-free survival (DFS), and identification of prognostic factors associated with poor outcomes. Additionally, we sought to characterize patients achieving long-term survival (OS ≥ 24 months). **Results:** The median OS and DFS were 18 and 13 months, respectively. A peritoneal cancer index (PCI) ≥ 7 and major postoperative complications were independently associated with reduced survival. Recurrence was significantly linked to PCI ≥ 7 and signet ring cell histology. Stratification by survival outcome identified PCI ≥ 7 as the only statistically significant variable differentiating average- and long-survival groups. Moreover, elevated PCI was independently associated with a higher incidence of major postoperative complications. **Conclusions:** CRS + HIPEC may offer a survival advantage over the use of systemic therapy exclusively in appropriately selected patients, particularly those with limited peritoneal disease burden. These results underscore the importance of accurate patient selection to balance surgical risks and maximize oncological benefits in the treatment of GCPM.

## 1. Introduction

Gastric cancer (GC) is the fifth most common malignancy and the fourth leading cause of cancer-related death worldwide. Diagnosis is often delayed, and peritoneal metastases (PM) are present at diagnosis in approximately 20–30% of cases [1,2], posing a major clinical challenge, with limited therapeutic options and poor prognosis.

The inadequate penetration of chemotherapeutic agents across the blood–peritoneal barrier and the high peritoneal tumor burden have traditionally limited the effectiveness of systemic chemotherapy, necessitating alternative treatment strategies [3,4,5].

To address this issue, multimodal treatment approaches—most notably the combination of cytoreductive surgery (CRS) with hyperthermic intraperitoneal chemotherapy (HIPEC)—have been introduced in recent years. However, the efficacy of both CRS and HIPEC remains uncertain, as current evidence is heterogeneous and often conflicting. The first randomized trial by Yang et al. (2011) reported improved overall survival with CRS + HIPEC compared to CRS alone [6]. While some subsequent studies and meta-analyses have suggested potential survival benefits in selected patients [7,8], these results have not been consistently replicated. For instance, the GASTRIPEC-I trial showed improvements in progression-free and metastasis-free survival, but no significant difference in overall survival between treatment arms [9]. More recent data from the REINASSANCE (AIO-FLOT5) trial suggest that patients treated with systemic chemotherapy alone may fare better in terms of overall survival than those undergoing surgical resection, raising further doubts about the benefit of aggressive locoregional strategies [10]. These findings reinforce the uncertainty surrounding the therapeutic value of CRS and HIPEC and underscore the importance of rigorous patient selection and accurate disease staging.

In this context, a multidisciplinary approach is essential to defining precise eligibility criteria, minimizing intraoperative and postoperative complications, and optimizing the balance between surgical aggressiveness and survival benefit.

The present study aims to evaluate the outcomes of CRS + HIPEC in patients affected by gastric cancer with peritoneal metastases (GCPM), with a specific focus on the identification of prognostic factors that negatively influence treatment outcomes. Furthermore, we sought to delineate the characteristics of a well-defined subgroup of patients who demonstrated long-term survival.

## 2. Materials and Methods

This retrospective, single-center study analyzed data from a prospectively maintained database of patients affected by synchronous GCPM undergoing CRS + HIPEC after neoadjuvant therapy at the Surgical Unit of Peritoneal and Retroperitoneal Surgery, Fondazione Policlinico Universitario Agostino Gemelli IRCCS, between January 2016 and September 2023. Patients with metachronous GCPM or a primary tumor located at the gastroesophageal junction (EGJ) were excluded. All the patients provided written informed consent and were enrolled in a follow-up program. The follow-up protocol included physical examinations, measurement of tumor marker levels (CEA, CA 125, CA 19.9), and regular radiological imaging (CT thorax and abdomen). The follow-up schedule was every 3 months in the first 2 years and every 6 months from the third year until 5 years after surgery.The relapse and survival data were collected and stored in a prospectively maintained database. This study was approved by the local institutional review board (Protocol No. 51892/19, ID 2923).

Following a thorough preoperative evaluation, all patients were assessed by our hospital’s multidisciplinary team specializing in peritoneal disorders. The extent of peritoneal involvement was determined through diagnostic laparoscopy, and the peritoneal carcinomatosis index (PCI) was documented for each case.

Patients underwent first-line chemotherapy regimens that included combinations of platinum-based drugs, with or without fluoropyrimidines and/or leucovorin, or a combination of platinum agents, with or without taxanes and fluoropyrimidines. Based on molecular profiling, biologic agents such as trastuzumab (for HER2-positive tumors) or nivolumab (for tumors with PD-L1 expression) were added to the chemotherapy regimen in selected cases. Following first-line chemotherapy, cross-sectional imaging was utilized to confirm no progression of peritoneal or extraperitoneal disease as per RECIST guidelines [11,12]. Patients exhibiting disease progression received second-line systemic chemotherapy. Conversely, those with no progression or stable disease and no evidence of other metastatic sites underwent a repeat diagnostic laparoscopy to re-evaluate the PCI score for surgical resectability.

The approach of the multidisciplinary team was to propose cytoreductive surgery combined with hyperthermic intraperitoneal chemotherapy (CRS + HIPEC) for patients with good performance status (ECOG < 2) who might achieve a complete cytoreduction of peritoneal disease in the absence of extra-abdominal metastases.

Patients who were not candidates for R0 resection due to the extent or location of the disease or due to comorbid conditions were considered for pressurized intraperitoneal aerosol chemotherapy (PIPAC).

### 2.1. Endpoints

The primary objective of this study was to evaluate overall survival (OS) and disease-free survival (DFS) in patients with carcinomatosis from gastric cancer (CGPM) treated with cytoreductive surgery (CRS) and hyperthermic intraperitoneal chemotherapy (HIPEC). We also aimed to identify predictive factors for unfavorable long-term outcomes. OS was defined from the date of CRS until death from any cause or censored at the last follow-up for surviving patients. DFS was defined from the date of CRS to the first documented recurrence.

A further aim was to characterize a subgroup of patients who achieved long-term survival. Patients with an OS of at least 24 months were considered the long-survival group (LS-G) if they died from disease progression after a minimum of 24 months or were still alive with 24 months of follow-up. Those who died from disease progression in less than 24 months were assigned to the average-survival group (AS-G). Patients who died from other causes or had fewer than 24 months of follow-up were excluded to minimize bias. The 24-month threshold was chosen based on published survival data and its proximity to the median OS in our series.

As a secondary objective, we assessed the intraoperative and short-term outcomes of CRS, including complications, 30-day mortality, and risk factors for postoperative morbidity.

### 2.2. Data

The clinical variables analyzed included age, gender, body mass index (BMI), Eastern Cooperative Oncology Group (ECOG) Performance Status [13], and American Society of Anesthesiologists (ASA) classification. Pathological variables assessed included primary tumor location (antrum vs. corpus/fundus), peritoneal cancer index (PCI) score [14], pathology stage (ypTNM) according to the 8th edition of the TNM classification (UICC/AJCC) [15], serosal invasion (classified as pT1-3 vs. pT4), node-positive status (classified as pN− vs. pN+), human epidermal growth factor receptor 2 (HER2) status, preoperative chemotherapy, signet ring cell histology, and Lauren classification (classified as intestinal vs. diffuse type). The surgical factors assessed included the type of gastrectomy and lymphadenectomy used, the extent of the peritonectomy, the need for multi-visceral resections, the completeness of the cytoreduction (CC-0, CC-1, CC-2), and the HIPEC drug administered post-surgery. The safety of the procedure was evaluated by analyzing intraoperative complications, postoperative complications classified according to the Clavien–Dindo classification [16], and 30-day mortality.

### 2.3. Surgical Technique

Following laparotomy, the PCI was re-evaluated; CRS + HIPEC was performed only if the extension of the peritoneal disease was deemed completely resectable. Cytoreductive surgery was performed, starting with the resection of the primary tumor and with total or subtotal gastrectomy associated with D2 or D2 + lymphadenectomy. Total gastrectomy was performed in all proximal tumors, and subtotal gastrectomy was performed in distal tumors if a 5–6 cm safety margin was present. Selective peritonectomy and multiple organ resections were performed, depending on disease extension, to achieve CC-0 resection [13]. HIPEC was performed using the closed technique. The HIPEC regimen consisted of a combination of cisplatin (75 mg/m^2^ in 250 mL NaCl) and mitomycin C (15 mg/m^2^ in 250 mL NaCl), and intravenous sodium thiosulfate was administered to prevent cisplatin-induced nephrotoxicity. The target temperature was set at 42 °C, and the time duration was 60 min. The adequate intra-abdominal patient filling volume was 2–2.5 L/m^2^.

### 2.4. Statistical Analysis

Continuous variables were presented as median (range, minimum–maximum). Categorical variables were reported as numbers and percentages (%). Multivariable analyses were conducted using multinomial logistic regression, integrating variables with univariate *p*-values ≤ 0.1. Kaplan–Meier curves were employed for the graphical illustration of the outcomes. The characteristics of AS-G and LS-G were compared using the Chi-square test or Fisher’s exact test for categorical variables, while the Mann–Whitney test or the Student’s *t*-test were adopted for continuous variables. Statistical significance was defined as *p*-values < 0.05, with 95% confidence intervals (CI). To further support the reliability of the findings, a post hoc statistical power analysis was conducted for all independent predictors identified in the multivariable models. All statistical analyses and graph generation were performed using GraphPad Prism 9 and IBM SPSS software v29.0.1.0.

## 3. Results

A total of 30 patients affected by gastric cancer with synchronous peritoneal metastases (GCPM) undergoing CRS + HIPEC were selected. The baseline characteristics and operative details are summarized in Table 1 and Table 2, respectively.

**Figure 1:**
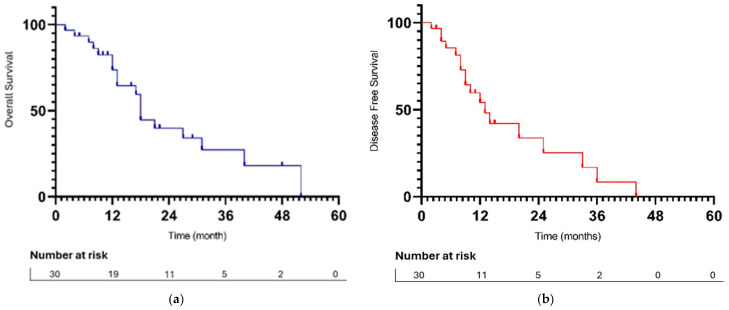
(**a**) OS from CRS + HIPEC; (**b**) DFS from CRS + HIPEC.

The median follow-up period was 23 months. All reported deaths were attributable to disease progression. The median overall survival (mOS) and disease-free survival (mDFS) from CRS were 18 months (range 2–52 months) and 13 months (range 2–44 months), respectively, for the entire study population (Figure 1).

A univariate analysis was performed, documenting factors associated with a worse prognosis PCI ≥ 7 (HR = 3.767, 95%CI [1.196–11.865], *p* = 0.023) and major post-operative complications (HR = 3.999, 95%CI [1.098–14.559], *p* = 0.036).

Multivariate analysis confirmed PCI ≥ 7 (HR = 4.258, 95%CI [1.325–13.682], *p* = 0.015) and major post-operative complications (HR = 5.047, 95%CI [1.261–20.203], *p* = 0.022) as independent factors that worsen prognosis (Table 3). The post hoc statistical power, calculated based on the observed hazard ratios (HR), total sample size (N = 30 patients), and α error set at 0.05, was 74.3% for PCI ≥ 7 and 83.7% for major post-operative complications, respectively.

Univariate analysis revealed a significant correlation between DFS and PCI ≥ 7 (HR = 2.824, 95%CI [1.203–9.731], *p* = 0.023), signet ring cells histology (HR = 3.167, 95%CI [1.139–8.803], *p* = 0.027), and diffuse type, according to the Lauren classification ((HR = 3.014, 95%CI [1.022–3.014], *p* = 0.046).

However, in the multivariate analysis, PCI ≥ 7 (HR = 5.975, 95%CI [1.412–10.286], *p* = 0.018) and signet ring cell histology (HR = 5.540, 95%CI [1.483–12.698], *p* = 0.011) emerged as the sole independent factors associated with poorer DFS (Table 4). The post hoc statistical power, calculated based on the observed hazard ratios (HR), total sample size (N = 30 patients), and α error set at 0.05, was 90.4.% for PCI ≥ 7 and 87.7% for signet ring cell histology, respectively.

**Figure 2:**
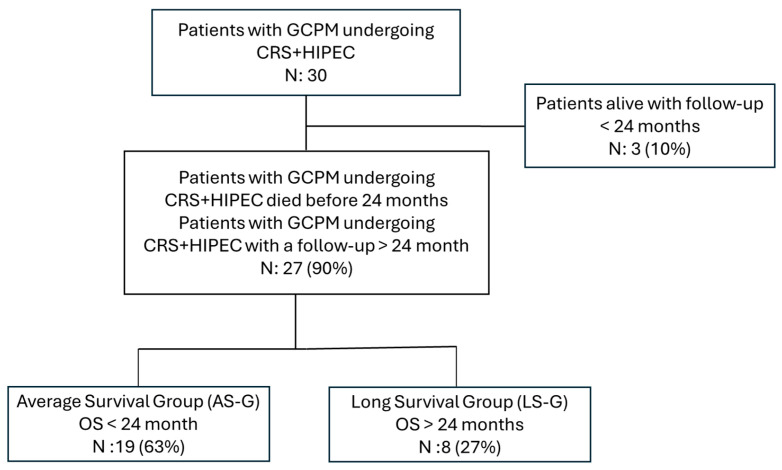
Flowchart of patient enrollment for long-survival analysis.

Among the entire cohort of 30 patients, 3 (10%) were excluded from further analysis because they were alive at the end of the observational period but had a follow-up period of less than 24 months. The remaining patients who met the inclusion criteria for the long-term survival analysis were divided into two groups using a 24-month survival threshold. The “average survival group” (AS-G: OS < 24 months from the date of surgery), consisted of 19 (63%) patients, and the “long-survival group” (LS-G: OS > 24 months from the date of surgery), consisted of 8 (27%) patients (Figure 2).

As shown in Table 5, the comparative analysis revealed that no statistically significant differences were found between the two groups for any of the variables analyzed, except for PCI ≥ 7, which was statistically more frequent in the AS-G (*p*-value 0.046).

Intraoperative complications occurred in four patients (13.3%), with four cases of diaphragmatic perforation. Postoperative complications of CD grade 3–4 were observed in seven patients (23.3%) and are summarized in Table 6. No 30-day mortality was observed.

Univariate analysis showed that major postoperative complications (CD 3–4) were correlated with PCI (OR = 1.221, 95% CI [1.089–1.328], *p* < 0.001) and blood loss > 500 mL (OR = 1.828, 95% CI [1.279-8.342], *p* = 0.032). Multivariate analysis identified PCI (OR = 1.196, 95% CI [1.023–1.251], *p* < 0.001) as the only independent predictor of major complications (Table 7).

## 4. Discussion

Despite the recent improvement in oncological outcomes achieved through the combination of chemotherapy and cytoreductive surgery, gastric cancer with peritoneal metastases (GCPM) remains a disease with a particularly poor prognosis [3,4,5].

In our study, survival outcomes following cytoreductive surgery (CRS) and hyperthermic intraperitoneal chemotherapy (HIPEC) are consistent with those reported in major case series [17,18] and exceed the survival rates previously observed among patients treated exclusively with chemotherapy [19,20].

The inherent selection bias in surgical cohorts, where patients with less extensive disease and good responses to chemotherapy are selected, complicates direct comparisons with chemotherapy-only groups, which often include patients with more advanced disease that are not treatable with surgery. The results from the REINASSANCE trial [21], presented at the 2024 ASCO Congress [10], further emphasize these concerns. In this trial, patients were randomized to receive either exclusive chemotherapy treatment or cytoreduction, in this way eliminating the selection bias. Accordingly, the surgical cohort exhibited a median overall survival of 12 months, which was notably lower than the 19 months observed in the chemotherapy-only group. Interestingly, in our study, the CRS cohort demonstrated a better median survival (17 months) compared to the 12 months observed in the surgical arm of the REINASSANCE trial. However, what is even more intriguing is that the chemotherapy-only arm of the REINASSANCE trial slightly outperformed our CRS group in terms of survival, raising significant doubts about the actual benefit and utility of CRS compared to chemotherapy alone.

Currently, one of the key factors determining the feasibility of CRS and guiding patient selection for surgical intervention is the PCI. The ideal PCI threshold for identifying oligometastatic disease which could benefit from aggressive local treatment remains unclear. However, lower PCI scores are associated with a higher likelihood of achieving complete cytoreduction, which is a crucial predictor of survival. While Glehen et al. proposed a PCI cut-off of 12, later studies have indicated that a PCI < 7 is linked to more favorable survival outcomes [22,23,24]. Our results are consistent with this prediction, as PCI ≥ 7 has been shown to correlate with early disease recurrence and reduced survival. Moreover, we analyzed patients whose survival exceeded the median in our cohort and identified a small subset of “long survivors”, with survival surpassing the overall median. A comparative analysis between these long survivors and the median-survival group revealed that the only statistically significant difference was the PCI. Notably, no patient with a PCI ≥ 7 was identified as a long survivor.

Although the small sample size limits the accuracy of our analysis, this finding suggests the existence of a restricted group of patients with a less-aggressive disease, characterized by confined peritoneal spread [25,26]. Such a clinical profile aligns with the “peritoneal oligometastatic disease” (OMD) concept, which describes an intermediate disease state marked by lower mutational burden, reduced aggressiveness, and a tendency toward local rather than systemic dissemination. Emerging evidence indicates that isolated peritoneal implants may represent an oligometastatic state in gastric cancer, offering a plausible explanation for the limited extent of peritoneal spread observed in some patients and their notable response to locoregional treatments [27].

However, beyond the ongoing uncertainties regarding the actual oncological benefits of cytoreductive surgery, which have been further emphasized by the recent results of the RENAISSANCE trial, it is crucial to carefully consider the rate of major complications associated with this procedure. In our cohort, the rate of major complications was 16.7%, consistent with those reported in other published studies [28,29,,]. Furthermore, our analysis revealed a significant correlation between higher PCI scores and an increased likelihood of experiencing major postoperative complications.

This underscores a crucial consideration regarding cytoreductive surgery: although it may provide modest survival benefits in patients with extensive peritoneal metastases, it also entails significant risks. In individuals with advanced disease or higher PCI scores, the potential benefits of surgery may be outweighed by postoperative complications, ultimately compromising long-term outcomes. In such scenarios, chemotherapy alone may represent a safer and more effective therapeutic option. Consequently, the decision to proceed with CRS requires careful consideration of both the potential benefits and the significant risks associated with higher PCI scores.

In this context, an encouraging finding from our study is the identification of a small subset of CRS patients who exhibit survival outcomes significantly exceeding the median survival reported in our cohort. Remarkably, these patients showed survival rates superior to those observed in the chemotherapy-only group of the RENAISSANCE trial [10]. This highlights the possibility of identifying a distinct group of patients who may derive substantial improvements in survival outcomes following CRS + HIPEC. Future studies should aim to identify factors that allow for the early classification of patients within this subgroup, enabling the development of more tailored treatment strategies. The identification of such patients is essential to optimize CRS selection criteria, ensuring that the procedure is reserved for those with the highest likelihood of benefit, thereby minimizing unnecessary risks in individuals less likely to respond favorably.

Therefore, it is essential to implement rigorous patient selection processes to optimize the balance between the risk of quality-of-life impairment due to surgical complications and the survival benefit. Given the steep learning curve associated with achieving proficiency in this technically demanding surgical procedure [30], it is our opinion that it should be performed exclusively in high-volume centers with specialized general surgery teams. These centers should adopt a collaborative approach involving other specialists, such as vascular, hepatobiliary, gynecologic, and urologic surgeons, as well as interventional radiologists, to reduce the risk of complications and optimize their management.

Given the absence of established guidelines for treating peritoneal carcinomatosis originating from gastric adenocarcinoma, the experiences of individual centers remain invaluable for shaping a consensus regarding best practices. Our study’s retrospective design and small patient cohort inevitably limit the conclusiveness of our findings. However, these constraints also offer a glimpse into real-world clinical practice, underscoring the need for larger, prospective, multicenter investigations. Despite these limitations, our data provide meaningful insights that may inform future efforts to refine treatment strategies and improve outcomes in patients with GCPM.

## 5. Conclusions

The role of CRS + HIPEC in treating carcinomatosis from gastric cancer, as well as its superiority over chemotherapy, currently remains controversial. However, a subgroup of patients undergoing surgical treatment achieves long-term survival. This finding underscores the need for further studies to identify the characteristics of this population, with the aim of refining patient selection and guiding them toward tailored treatment strategies to enhance oncological outcomes.

## Figures and Tables

**Table 1:** Population baseline characteristics.

Variable	CRS
	30 Patients
**Age median (Min–Max)**	56 (26–74)
**Gender, n (%)**	
Male	12 (40)
Female	18 (60)
**BMI median (Min–Max)**	23.89 (19–38)
**ASA, n (%)**	
Score < 3	26 (86.7)
Score ≥ 3	4 (13.3)
**ECOG n (%)**	
Grade 0	17 (56.7)
Grade ≥ 1	13 (43.3)
**Tumor location, n (%)**	
Antrus	8 (26.7)
Fundus/corpus	22 (73.3)
**Signet ring cell histology, n (%)**	
Yes	9 (30)
No	21 (70)
**Lauren classification, n (%)**	
Intestinal	19 (63.3)
Diffuse	11 (36.7)
**Median PCI at staging laparoscopy (Min–Max)**	5.5 (1–14)
**Median PCI at cytoreduction (Min–Max)**	
**T status, n (%)**	
1	-
2	-
3	11 (36–7)
4	19 (63.3)
**Nodal status, n (%)**	
0	3 (10)
1	5 (16.7)
2	8 (26.7)
3	14 (46.7)
**HER-2 overexpression, n (%)**	
Positive	3 (10)
Negative	27 (90)
**Neoadjuvant chemotherapy ± biological agents**	30 (100)
Fluoropyrimidine + oxaliplatin/cisplatin ± leucovorin	18 (60)
Docetaxel + fluoropyrimidine + oxaliplatin/cisplatin	12 (40)
**Adjuvant chemotherapy**	19 (63.3)

**Table 2:** Operative details in patients undergoing CRS + HIPEC.

Variable	CRS
	30 Patients
**Median operative time, min (Min–Max)**	490 [240–750]
**Median PCI at cytoreduction**	3.5 (0–12)
**Peritoneal complete response**	5 (16.6)
**Type of gastrectomy, n (%)**	
Total gastrectomy	19 (63.3)
Subtotal gastrectomy	11 (36.7)
**Type of lymphadenectomy, n (%)**	
D2	24 (80)
D2 +	6 (20)
**Additional procedure, n (%)**	
Colon resection	5 (16.7)
Rectal resection	7 (23.3)
Pelvic Peritonectomy	8 (26.7)
Diaphragmatic peritonectomy	14 (46.7)
Hysterectomy/oophorectomy	6 (20)
Small bowel resection	3 (10)
Splenectomy	11 (36.7)
Ostomy	3 (10)
**CCR, n (%)**	
Complete	29 (96.7)
Incomplete	1 (3.3)
**HIPEC, n (%)**	
Yes	30 (100)
No	-
**HIPEC drug, n (%)**	
Cisplatin + mitomicyn C	30 (100)

**Table 3:** Multivariate analysis of factors influencing OS in patients undergoing CRS+HIPEC.

Variables		Univariate Analysis			Multivariate Analysis	
	HR	95% CI	*p*-Value	HR	95%CI	*p*-Value
**Age**	1.036	[0.984–1.092]	0.180			
**Gender**						
Male	0.509	[0.175–1.478]	0.214			
Female						
**BMI**	0.822	[0.803–1.216]	0.197			
**ECOG ≥ 1**	1.095	[0.510–2.352]	0.815			
**ASA ≥ 3**	2.892	[0.695–3.398]	0.092	1.500	[0.685–3.283]	0.311
**PCI ≥ 7**	3.767	[1.196–11.865]	0.023	4.258	[1.325–13.682]	0.015
**Lymph-nodes +**	1.255	[0.404–2.008]	0.482			
**pT4**	1.585	[0.539–4.668]	0.403			
**HER-2 overexpression**	2.430	[0.623–9.482]	0.201			
**Tumor location**						
Antrus	1.373	[0.461–4.095]	0.568			
Fundus/corpus						
**Signet ring cell histology**						
Yes	1.742	[0.528–5.750]	0.362			
No						
**Lauren classification**						
Diffuse	2.106	[0.672–2.759]	0.507			
Intestinal						
**Splenectomy**	1.743	[0.209–14.512]	0.608			
**Colorectal resection**	0.450	[0.099–2.032]	0.299			
**Hysterectomy/oophorectomy**	1.551	[0.549–4.387]	0.408			
**Pelvic peritonectomy**	1.578	[0.529–4.705]	0.413			
**Diaphragmatic peritonectomy**	2.519	[0.761–8.338]	0.130			
**Small bowel involvement**	2.250	[0.483–10.477]	0.301			
**Major post-operative complication**	3.999	[1.098–14.559]	0.036	5.047	[1.261–20.203]	0.022
**Intra-operative complication**	2.045	[0.556–7.516]	0.282			
**Neoadjuvant chemotherapy**						
−Docetaxel + fluoropyrimidine + oxaliplatin/cisplatin	0.832	0.648–1.276	0.715			
−Fluoropyrimidine + oxaliplatin/cisplatin ± leucovorin						
**Neoadjuvant chemotherapy > 4 cycles**	0.548	0.25–1.22	0.198			
**Adjuvant chemotherapy**	0.726	0.32–1.63	0.439			

Statistical significance (*p* < 0.05): statistically significant values are underlined.

**Table 4:** Multivariate analysis of factors influencing DSF in patients undergoing CRS + HIPEC.

Variables		Univariate Analysis			Multivariate Analysis	
	HR	95% CI	*p*-Value	HR	95%CI	*p*-Value
**Age**	0.997	[0.941–1.057]	0.937			
**Gender**						
Male	0.766	[0.279–2.129]	0.609			
Female						
**BMI**	0.903	[0.705–1.157]	0.420			
**ECOG ≥ 1**	3.150	[0.689.9.459]	0.116			
**ASA ≥ 3**	1.506	[0.845–3.769]	0.313			
**PCI ≥ 7**	2.824	[1.203–9.731]	0.023	5.975	[1.412–10.286]	0.018
**Lymph-nodes +**	1.011	[0.977–1.046]	0.534			
**pT4**	1.510	[0.507–4.479]	0.460			
**HER-2 overexpression**	2.862	[0.567–14.442]	0.203			
**Tumor location**						
Antrus	0.572	[0.154–2.219]	0.405			
Fundus/corpus						
**Signet ring cell histology**						
Yes	3.167	[1.139–8.803]	0.027	5.540	[1.483–12.698]	0.011
No						
**Lauren classification**						
Diffuse	3.014	[1.022–3.014]	0.046	2.077	[0.643–6.711]	0.222
Intestinal						
**Splenectomy**	1.351	[0.280–6.511]	0.708			
**Colorectal resection**	2.344	[0.601–9.146]	0.220			
**Hysterectomy/oophorectomy**	2.507	[0.711–5.956]	0.184			
**Pelvic peritonectomy**	1.934	[0.649–5.672]	0.236			
**Diaphragmatic peritonectomy**	3.129	[0.770–12.722]	0.111			
**Small bowel involvement**	1.999	[0.694–5.756]	0.200			
**Major post-operative complication**	2.987	[0.440–3.149]	0.182			
**Intra-operative complication**	2.016	[0.416–9.762]	0.384			
**Neoadjuvant chemotherapy**						
−Docetaxel + fluoropyrimidine + oxaliplatin/cisplatin	0.692	[0.186–1381]	0.274			
−Fluoropyrimidine + oxaliplatin/cisplatin ± leucovorin						
**Neoadjuvant chemotherapy > 4 cycles**	0.581	0.33–1.42	0.212			
**Adjuvant chemotherapy**	0.711	0.30–1.45	0.328			

Statistical significance (*p* < 0.05): statistically significant values are underlined.

**Table 5:** Statistical comparative analysis between AS-G and LS-G.

Variable	Short-Survival Group (S.SG): 19 pz	Long-Survival Group (L-SG): 8 pz	*p*-Value
**Age**	56 (26–73)	57.5 (47–74)	0.193
**Gender**			0.495
Male	8 (42.1)	4 (50)	
Female	11 (57.9)	4 (50)	
**BMI**	24 (19–38)	24.5 (22–27)	0.572
**ECOG ≥ 1**	10 (53.6)	3 (37.5)	0.474
**ASA ≥ 3**	3 (15.8)	1 (12.5)	0.826
**PCI ≥ 7**	7 (36.8)	0 (0)	0.046
**pT4**	10 (52.6)	3 (37.5)	0.233
**HER-2 overexpression**	3 (15.8)	0 (0)	0.271
**Tumor location**			0.561
Antrus	5 (26.3)	2 (25)	
Fundus/corpus	14 (73.7)	6 (75)	
**Signet ring cells**			0.400
Yes	8 (42.1)	2 (25)	
No	11 (57.9)	6 (75)	
**Lauren**			0.365
Intestinal	6 (31.6)	4 (50)	
Diffuse	13 (68.4)	4 (50)	
**Splenectomy**	6 (31.6)	2 (25)	0.732
**Colorectal resection**	3 (15.8)	0 (0)	0.233
**Hysterectomy/oophorectomy**	4 (21.1)	1 (12.5)	0.601
**Pelvic peritonectomy**	6 (31.6)	2 (25)	0.900
**Diaphragmatic peritonectomy**	11 (50)	3 (37.5)	0.685
**Small bowel involvement**	3 (15.8)	0 (0)	0.271
**Major post-operative complication**			0.397
Yes	5 (26.3)	1 (12.5)	
No	14 (73.7)	7 (87.5)	
**Intra-operative complication**			0.826
Yes	3 (15.8)	1 (12.5)	
No	16 (84.2)	7 (87.5)	

Statistical significance (*p* < 0.05): statistically significant values are underlined.

**Table 6:** Intraoperative and postoperative complications.

Variable	CRS 30 Patients
**Intra-operative complication, n (%)**	
Yes	4 (13.3)
No	26 (86.7)
**Types of Intraoperative complication, n (%)**	
Diaphragmatic laceration	4 (13.3)
**Postoperative complication, n (%)**	
Yes	15 (50)
No	
**Clavien–Dindo grade, n (%)**	
I	5 (16.6)
II	3 (10)
IIIa	4 (13.3)
IIIb	2 (6.6)
IV	1 (3.3)
V	-
**Types of post-operative complication, n (%)**	
Post-operative ileus	3 (10)
Wound infection	1 (3.3)
Sepsis	1 (3.3)
Anemia	2 (6.6)
Abdominal collection	4 (13.3)
Anastomotic bleeding	1 (3.3)
Hemoperitoneum	1 (3.3)
Pleural effusion	1 (3.3)
Septic shock	1 (3.3)
**30-day mortality**	-

**Table 7:** Multivariate analysis of factors associated with major postoperative complications in patients undergoing CRS + HIPEC.

Variables	Univariate Analysis			Multivariate Analysis		
	OR	95% CI	*p*-Value	OR	95% CI	*p*-Value
**Age**	1.009	[0.929–1.095]	0.839			
**Gender**						
Male	1.667	[0.275–8.234]	0.578			
Female						
**BMI**	0.837	[0.579–1.210]	0.344			
**ECOG ≥ 1**	1.625	[0.859–9.921]	0.201			
**ASA ≥ 3**	1.222	[0.390–2.285]	0.223			
**Primary tumor location**						
Antrus	0.667	[0.061–7.230]	0.793			
Fundus/corpus						
**Signet ring cells**						
Yes	1.905	[0.150–6.671]	0.998			
No						
**Lauren**						
Intestinal						
Diffuse	2.600	[0.394–9.562]	0.321			
**PCI**	1.221	[1.089–1.328]	< 0.001	1.196	[1.023–1.251]	<0.001
**Type of gastrectomy**						
Total	1.343	[0.384–5.329]	0.563			
Sub-total						
**Surgical procedure**						
Splenectomy	0.792	[0.048–4.945]	0.542			
Colorectal resection	2.333	[0.414–14.523]	0.258			
Bowel resection	1.417	[0.204–9.817]	0.724			
Hysterectomy/oophorectomy	1.256	[0.184–9.660]	0.776			
Diaphragmatic peritonectomy	0.860	[0.072–8.067]	0.820			
Pelvic peritonectomy	1.000	[0.150–6.671]	1.000			
Ostomy	0.217	[0.012–4.094]	0.308			
**Blood loss > 500 mL**						
Yes	1.828	[1.279–8.342]	0.032	1.176	[0.593–6.698]	0.783
No						
**Intra-operative complications**						
Yes	1.400	[0.119–16.459]	0.789			
No						

Statistical significance (*p* < 0.05): statistically significant values are underlined.

## Data Availability

The raw data supporting the conclusions of this article will be made available by the authors on request.

## Abbreviations

The following abbreviations are used in this manuscript:

AJCC	American Joint Committee on Cancer
AS-G	Average-Survival Group
ASA	American Society of Anesthesiologists
CC	Completeness of Cytoreduction Score
CD	Clavien–Dindo Classification of Complications
CI	Confidence Interval
CRS	Cytoreductive Surgery
DFS	Disease-Free Survival
ECOG	Eastern Cooperative Oncology Group (Performance Status)
GC	Gastric Cancer
GCPM	Gastric Cancer with Peritoneal Metastases
HER2	Human Epidermal Growth Factor Receptor 2
HIPEC	Hyperthermic Intraperitoneal Chemotherapy
LS-G	Long-Survival Group
mDFS	Median Disease-Free Survival
mOS	Median Overall Survival
OMD	Oligometastatic Disease
OR	Odds Ratio
OS	Overall Survival
PCI	Peritoneal Cancer Index
PIPAC	Pressurized Intraperitoneal Aerosol Chemotherapy
PM	Peritoneal Metastases
UICC	Union for International Cancer Control
UOC	Complex Operative Unit
